# Combined hyperglycemic crises in adult patients already exist in Latin America.

**DOI:** 10.7705/biomedica.6912

**Published:** 2024-05-31

**Authors:** Guillermo Edinson Guzmán, Veline Martínez, Sebastián Romero, María Mercedes Cardozo, María Angélica Guerra, Oriana Arias

**Affiliations:** 1 Departamento de Endocrinología, Fundación Valle del Lili, Cali, Colombia Departamento de Endocrinología Fundación Valle del Lili Cali Colombia; 2 Departamento de Medicina Interna, Fundación Valle del Lili, Cali, Colombia Departamento de Medicina Interna Fundación Valle del Lili Cali Colombia; 3 Facultad de Ciencias de la Salud, Universidad ICESI, Cali, Colombia Universidad Icesi Facultad de Ciencias de la Salud Universidad ICESI Cali Colombia; 4 Centro de Investigaciones Clínicas, Fundación Valle del Lili, Cali, Colombia Centro de Investigaciones Clínicas Fundación Valle del Lili Cali Colombia

**Keywords:** Hyperglycemia, diabetic ketoacidosis, diabetes mellitus, mortality, Latin America, hiperglucemia, cetoacidosis diabética, diabetes mellitus, mortalidad, América Latina

## Abstract

**Introduction.:**

Diabetes mellitus is one of the most common diseases worldwide, with a high morbidity and mortality rate. Its prevalence has been increasing, as well as its acute complications, such as hyperglycemic crises. Hyperglycemic crises can present with combined features of diabetic ketoacidosis and hyperosmolar state. However, their implications are not fully understood.

**Objective.:**

To describe the characteristics, outcomes, and complications of the diabetic population with hyperglycemic crises and to value the combined state in the Latin American population.

**Materials and methods.:**

Retrospective observational study of all hyperglycemic crises treated in the intensive care unit of the *Fundación Valle del Lili* between January 1, 2015, and December 31, 2020. Descriptive analysis and prevalence ratio estimation for deaths were performed using the robust Poisson regression method.

**Results.:**

There were 317 patients with confirmed hyperglycemic crises, 43 (13.56%) with diabetic ketoacidosis, 9 (2.83%) in hyperosmolar state, and 265 (83.59%) with combined diabetic ketoacidosis and hyperosmolar state. Infection was the most frequent triggering cause (52.52%). Fatalities due to ketoacidosis occurred in four patients (9.30%) and combined diabetic ketoacidosis/hyperosmolar state in 22 patients (8.30%); no patient had a hyperosmolar state. Mechanical ventilation was associated with death occurrence (adjusted PR = 1.15; 95 % CI 95 = 1.06 - 1.24).

**Conclusions.:**

The combined state was the most prevalent presentation of the hyperglycemic crisis, with a mortality rate similar to diabetic ketoacidosis. Invasive mechanical ventilation was associated with a higher occurrence of death.

## Resumen

**Introducción.:**

La diabetes mellitus es una de las enfermedades más frecuentes en todo el mundo, con una tasa elevada de morbimortalidad. Su prevalencia ha ido en aumento y, también, sus complicaciones agudas, como las crisis hiperglucémicas. Las crisis hiperglucémicas pueden presentar características combinadas de cetoacidosis diabética y estado hiperosmolar. Aún no se conocen completamente sus implicaciones.

**Objetivo.:**

Describir las características, los resultados y las complicaciones de la población diabética con crisis hiperglucémicas, y valorar el estado mixto en la población latinoamericana.

**Materiales y métodos.:**

Se trata de un estudio observacional retrospectivo de pacientes con crisis hiperglucémicas atendidos en la unidad de cuidados intensivos de la Fundación Valle del Lili, entre el 1° de enero de 2015 y el 31 de diciembre de 2020. Se realizó un análisis descriptivo y se estimó la razón de prevalencia para muerte mediante el método de regresión de Poisson.

**Resultados.:**

Se incluyeron 317 pacientes con crisis hiperglucémica confirmada, 43 (13,56 %) con cetoacidosis diabética, 9 (2,83 %) en estado hiperosmolar y 265 (83,59 %) en estado mixto. La causa desencadenante más frecuente fue la infección (52,52 %). Cuatro pacientes fallecieron por cetoacidosis (9,30 %), 22 (8,30 %), por un estado mixto; ninguno se encontraba en estado hiperosmolar. La asistencia respiratoria mecánica se asoció con la muerte (razón de prevalencia ajustada = 1,15; IC_95%_: 1,06-1,24).

**Conclusiones.:**

El estado combinado fue la presentación más prevalente de la crisis hiperglucémica, con una tasa de mortalidad similar a la de la cetoacidosis diabética, y la asistencia respiratoria mecánica invasiva se asoció con una mayor ocurrencia de muerte.

Diabetes mellitus is one of the most common chronic non-communicable diseases, with high morbidity and mortality. Its prevalence is constantly increasing, with currently more than 463 million people worldwide suffering from diabetes ^1^^,^^2^. Approximately 8% of the Colombian population has this disease ^3^^,^^4^. It has a wide spectrum of clinical presentations ranging from silent with long-term consequences to acute manifestations with a major impact on a patient’s health status. Among the latter are diabetic ketoacidosis, hyperosmolar hyperglycemic state, and combined states, all of which are acute complications that lead to an increase in emergency consultation, hospital admission, and intensive care unit management ^5^.

As the prevalence of diabetes increases in the general population, so does the hyperglycemic crises; diabetic ketoacidosis is the most common form of crisis and is more frequent in young patients with type 1 diabetes ^6^^,^^7^. The hyperosmolar state occurs almost exclusively in patients with type 2 diabetes, and mortality in this group is higher when compared to diabetic ketoacidosis (5-20% vs. 1-5%) ^5^^,^^8^^,^^9^. Most studies describe hyperglycemic crises separately, limiting the analysis of combined forms ^8^^,^^10^. However, these cases have increased in prevalence in the last few years, as the literature shows that the mortality rate in the combined forms is higher than in the isolated cases (diabetic ketoacidosis, hyperosmolar state), even in the presence of severe hypokalemia and hypoglycemia ^11^.

This study aimed to describe the diabetic population with isolated or combined hyperglycemic crises admitted by the emergency department and managed at *Fundación Valle del Lili*, a high-complexity university hospital and reference center located in southwestern Colombia.

## Materials and methods

### Study design

A retrospective descriptive observational study was conducted at *Hospital Universitario Fundación Valle del Lili* in Cali, Colombia, between January 1, 2015, and December 31, 2020. The data were retrieved from the medical records department and the clinical record system (SAP) of the clinic with the approval of the institutional ethical committee. The patients were selected from the clinical record system and were diagnosed with hyperglycemic crises using the codes of the International Classification of Diseases (IDC-10) (E10.0 to E14.9).

### Participants and data collection

Participants were aged 18 years or older, diagnosed with hyperglycemic crises, admitted to the emergency room, and hospitalized in the intensive care unit. Patients who met the following criteria were included: glycemia higher than 250 mg/dl, ketones in serum or urine, and metabolic acidosis (pH < 7.3, HCO_3_ > 18 mEq/l) at admission for the case of diabetic ketoacidosis; plasma osmolarity greater than or equal to 320 mOsm/kg or plasma glucose greater than 600 mg/dl plus serum bicarbonate higher than 18 mEq/l for the hyperglycemic hyperosmolar state. In addition to meeting diabetic ketoacidosis criteria, if they had plasma osmolarity higher than or equal to 320 mOsm/kg, the patient was classified as a combined diabetic ketoacidosis-hyperosmolar state case ^12^^,^^13^. Medical records of minors and pregnant patients were excluded.

For all patients, we retrieved demographic data: age, sex, and medical history, such as arterial hypertension, coronary heart disease, and chronic kidney disease. Regarding diabetes mellitus, type, type of crisis, triggering cause, and disease control according to HbA1c were collected, as well as physical examination variables such as weight, height, and body mass index (BMI).

The paraclinical tests were evaluated at the time of admission to the intensive care unit, including complete blood count, renal function, electrolytes, arterial blood gasses, and plasma osmolarity. As outcomes, the need for invasive mechanical ventilation (IMV), days of overall hospital stay, presence of hypoglycemia, and mortality were recorded.

### Statistical analysis

Data were analyzed using Stata, version 15.0, software. A descriptive analysis of the variables was performed and presented according to their distribution. The factors associated with mortality were identified using Poisson regression. Normality was checked using the Shapiro-Wilk test. Normally distributed data are presented as the mean and standard deviation; non-normally distributed data are presented as the median and interquartile.

### Ethical aspects

The protocol application was approved by the Ethics Committee of the *Hospital Universitario Fundación Valle del Lili*. Informed consent was not obtained, given the retrospective character of the study.

## Results

**Figure 1.**
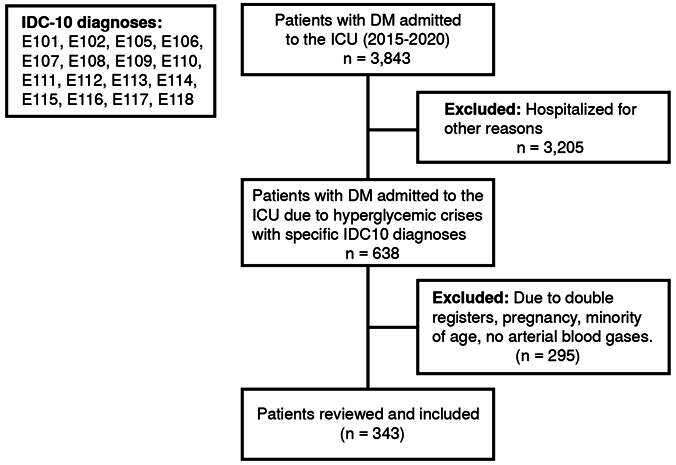
Flowchart of the included patients (ICD-10: International Classification of Diseases 10)

DM: Diabetes mellitus; ICU: Intensive care unit

Among 3,843 patients admitted to the intensive care unit, 638 were initially eligible for the study, 321 were excluded, including 317 for analysis with 43 diabetic ketoacidosis (13.56%), 9 hyperosmolar state (2.84%), and 265 combined diabetic ketoacidosis-hyperosmolar state (83.6%) (figure 1). There were 272 type 2 diabetes mellitus patients (88.80 %) and 39 diabetic ketoacidosis patients (90.70%) (table 1).

E10.1 Insulin-dependent diabetes mellitus, with coma; E10.2 Insulin-dependent diabetes mellitus, with ketoacidosis; E10.5 Insulin-dependent diabetes mellitus, with neurological complications; E10.6 Insulindependent diabetes mellitus, with peripheral circulatory complications; E10.7 Insulin-dependent diabetes mellitus, with other specified complications; E10. 8 Insulin-dependent diabetes mellitus, with multiple complications; E10.9 Insulin-dependent diabetes mellitus, with unspecified complications; E11.0 Noninsulindependent diabetes mellitus; E11.1 Noninsulin-dependent diabetes mellitus, with coma; E11.2 Noninsulindependent diabetes mellitus, with ketoacidosis; E11.3 Noninsulin-dependent diabetes mellitus, with renal complications; E11. 4 Noninsulin-dependent diabetes mellitus, with ophthalmic complications; E11.5 Noninsulin-dependent diabetes mellitus, with neurological complications; E11.6 Noninsulin-dependent diabetes mellitus, with peripheral circulatory complications; E11.7 Noninsulin-dependent diabetes mellitus, with other specified complications; E11.8 Noninsulin-dependent diabetes mellitus, with multiple complications.

**Table 1:** General characteristics of patients with hyperglycemic crises

Characteristics		General (N = 317)	DKA (n = 43)	HHS (n = 9)	Combined DKA-HHS (n = 265)	p value
Age (years)*		64 (48-71)	67 (65-70)	67 (65-70)	60 (44-70)	0.08
Year of attention [n (%)]						
	2012	1 (0.31)	0 (0)	0 (0)	1 (0.36)	
	2013	1 (0.31)	0 (0)	0 (0)	1 (0.36)	
	2014	2 (0.63)	0 (0)	0 (0)	2 (0.71)	
	2015	30 (9.43)	2 (4.65)	0 (0)	28 (10.56)	
	2016	59 (18.55)	6 (13.95)	1 (11.11)	52 (19.62)	
	2017	35 (11.01)	8 (18.60)	2 (22.22)	25 (9.43)	
	2018	36 (11.32)	7 (16.27)	0 (0)	29 (10.94)	
	2019	52 (16.35)	4 (9.30)	3 (33.33)	45 (16.98)	
	2020	102 (32.08)	16 (37.20)	3 (33.33)	82 (30.94)	
Sex: men [n (%)]		151 (47.48)	17 (39.53)	3 (33.33)	131 (49.43)	0.33
Diabetes [n (%)]						
	Type 1	38 (11.99)	3 (6.98)	0 (0)	35 (13.21)	0.57
	Type 2	272 (85.80)	39 (90.70)	9 (100)	224 (84.53)	
	Other	7 (2.21)	1 (2.33)	0 (0)	6 (2.26)	
Hypertension [n (%)]		171 (53.94)	26 (60.47)	7 (77.78)	138 (52.08)	0.20
Coronary heart disease [n (%)]		46 (14.51)	6 (13.95)	2 (22.22)	38 (14.34)	0.79
Chronic kidney disease[n (%)]		61 (19.24)	6 (13.95)	4 (44.44)	51 (19.25)	0.10

DKA: Diabetic ketoacidosis; HHS: Hyperosmolar hyperglycemic state.

Values are reported as absolute numbers (percentages).

* Reported as median (interquartile range)

In diabetic ketoacidosis, the mean age was 64 years, pH = 7.2 (7.047.24), bicarbonate = 11.4 mEq/L (6.9-15.1), glucose = 416 mg/dl (365-533) and osmolarity = 315 mOsm/L (303-332). For hyperosmolar state, the mean age was 67 years (65-70), pH = 7.37 (7.33-7.41), bicarbonate = 21.2 mEq/L (19.9-21.8), glucose = 685 mg/dl (618-823), and osmolarity = 342 mOsm/L (330-386). For combined diabetic ketoacidosis-hyperosmolar state, the mean age was 60 years (44-70), pH = 7.33 (7.19-7.40), bicarbonate = 15.85 mEq/L (10.7- 9.65), glucose = 467 mg/dl (356-500), and osmolarity = 314 mOsm/L (304-330) (table 2).

**Table 2:** Laboratory data of patients with hyperglycemic crises

	General (N = 317)	DKA (n = 43)	HHS (n = 9)	Combined DKA-HHS (n = 265)	p value
Hemoglobin** (g/dl)	12.4	12.6	11.8	12.3	0.80
	(10.3-14.2)	(10.1-13.9)	(10.9-13)	(10.2-14.3)	
Leukocytes* (u/L)	12,260	13,720	11,190	12,050	0.14
	(8080-17810)	(8850-21830)	(7720-14140)	(7760-17720)	
Creatinine* (mg/dl)	1.33	1.43	1.55	1.29	0.06
	(0.92-2.06)	(1.06-3.10)	(1.14-3.30)	(0.89-1.91)	
BUN* (mg/dl)	30.05	29.8	32.0	30.05	0.95
	(19.8-49.75)	(20.9-47.3)	(18.6-65.9)	(19.8-49.9)	
Sodium* (mmol/L)	136	137	144	136	0.14
	(132-141)	(133-141)	(136-148)	(132-141)	
Potassium* (mmol/L)	4.55	4.73	4.31	4.54	0.23
	(3.97-5.1)	(4.20-5.33)	(3.86-4.91)	(3.91-5.08)	
Chlorine* (mmol/L)	102	104	106	102	0.55
	(96.95-108)	(98.8-108)	(96-113)	(96.9-108)	
Bicarbonate** (mEq/L)	15.5	11.4	21.2	15.85	0.00
	(9.9-19)	(6.9-15.1)	(19.9-21.8)	(10.7-19.65)	
Arterial pH	7.31	7.2	7.37	7.33	0.00
	(7.16-7.39)	(7.04-7.24)	(7.33-7.41)	(7.19-7.40)	
HbA1c%	11.37	11.7	11	11.4	0.62
	(9.34-13.4)	(7.2-13.2)	(10.33-11.67)	(9.6-13.5)	
Plasma osmolarity** (mOsm/L)	314	315	342	314	0.016
	(304-332)	(303-332)	(330-386)	(304-330)	
Glycemia on admission**	470	416	685	467	0.00
	(360-525.7)	(365-533)	(618-823)	(356-500)	
Ketonuria [n (%)]	82	20	4	58	0.00
	(34,75)	(24,39)	(4,88)	(70,73)	
Anion gap** (mEq/L)	24	25	18	23	0.00
	(19-29)	(22-30)	(15-25)	(18-29)	

DKA: diabetic ketoacidosis; HHS: hyperosmolar hyperglycemic state; BUN: blood urea nitrogen; HbA1c: glycated hemoglobin Values are reported as absolute numbers (percentages).

* Reported as the median (interquartile range)

** Reported as the mean (standard deviation)

In general, the most frequent precipitating cause was infection, followed by poor adherence to treatment independent of the crisis type.

### Hospital length of stay, complications and mortality

The duration of hospitalization, measured by the time in the intensive care unit, had a median of four days; the longest was caused by combined diabetic ketoacidosis-hyperosmolar state with five days (p = 0.512). Hypoglycemia during insulin infusion was seen in 55 patients (17.35%): 9 with diabetic ketoacidosis (20.03%), 45 with combined diabetic ketoacidosis-hyperosmolar state (42.02%), and 2 with hyperosmolar state (22.2%) (p < 0.04).

Mechanical ventilation was required in 45.74% of the cases and was particularly common in diabetic ketoacidosis (67.44%). During hospitalization, a total of 26 deaths related to hyperglycemic crises were reported (8.20%), with more deaths in the diabetic ketoacidosis group (n = 6; 13.95%), followed by the diabetic ketoacidosis-hyperosmolar state group (n = 20, 7.55%). No mortal cases were reported in the hyperosmolar state (table 3).

**Table 3.** Outcomes of patients with hyperglycemic crises

Characteristics	General (N = 317)	DKA (n = 43)	HHS (n = 9)	Combined DKA-HHS (n = 265)	p value
ICU length of stay	4 (2-9)	6 (2-9)	4 (3-5)	4 (2-9)	0.790
Precipitating cause, n (%) Poor adherence to treatment	71 (22.40)	7 (16.28)	3 (33.33)	61 (23.02)	0.09 -
Infection	167 (52.68)	23 (53.49)	3 (33.33)	141 (53.21)	-
Acute myocardial Infarction	14 (4.42)	3 (6.98)	0 (0)	11 (4.15)	-
Stroke	4 (1.26)	0 (0)	0 (0)	4 (1.51)	-
Other	61 (19.24)	10 (23.26)	3 (33.33)	48 (18.11)	-
Hypoglycemia during insulin infusion*	55 (17.35)	9 (20.93)	1 (11.11)	45 (16.98)	0.04
Invasive mechanical ventilation	145 (45.74)	29 (67.44)	2 (22.22)	114 (43.02)	0.18
[n (%)] Death in hospital related to	26 (8.20)	6 (13.95)	0 (0)	20 (7.55)	0.09
hyperglycemic crises [n (%)]					

ICU: intensive care unit; DKA: diabetic ketoacidosis; HHS: hyperosmolar hyperglycemic state

Values are reported as absolute numbers (percentages).

* Reported as median (interquartile range)

**Reported as the mean (standard deviation)

During the analysis of the different risk factors related to mortality, we found that mortality in patients with hyperglycemic crises, adjusted by age and sex, was not determined by factors such as the type of diabetes, precipitating cause, osmolarity, blood urea nitrogen, or hemoglobin (adjusted PR = 1.00; 95% IC = 1.006-1.009). However, invasive mechanical ventilation (adjusted PR = 6.53; 95% IC = 2.29-18.54) and pH < 7.3 (adjusted PR = 2.33; 95% IC = 1.04-5.22) increased mortality rate (table 4).

**Table 4:** Adjusted prevalence ratios for mortality of patients with hyperglycemic crises

Characteristic		Adjusted RP	95 % CI
Age > 65 years		1.39	0.66-2.91
Male		1.01	0.95-1.09
Cause			
	Infection	1.67	0.76-3.64
	Myocardial Infarction	1.79	0.46-6.85
	Stroke	1.05	0.97-1.15
	Diabetes type 2	0.96	0.88-1.05
	Invasive mechanical ventilation	6.53	2.29-18.54
	Hypoglycemia	1.41	0.59-3.37
	BUN > 24.75 mmol/L	0.95	0.88-1.02
	Hb < 10 mg/dl	1.01	0.93-1.09
	pH < 7.3	2.33	1.04-5.22
	HCO_3_ < 18	1.31	0.54-3.17
	Osmolarity > 320	1.00	1.006-1.009

PR: prevalence ratio; CI: confidence interval; BUN: blood urea nitrogen; Hb: hemoglobin; HCO_3_: bicarbonate

## Discussion

Diabetes is a highly prevalent disease worldwide that generates acute and chronic complications, including diabetic ketoacidosis and a hyperosmolar hyperglycemic state, both with high morbidity and mortality rates as well as high costs for the health system ^14^. Diabetes mellitus treatment has changed; however, the prevalence of complications is high, as is their impact. Our study is one of the largest about hyperglycemic crises, comprising population characteristics and outcomes in terms of hospitalization length in days and mortality, and shows how the combined diabetic ketoacidosis/ hyperosmolar state is more frequent in contrast to other publications, even using the same classification criteria ^7^^,^^15^. Observational studies suggest that coexisting conditions could be an option, but management strategies are unclear. The hyperosmolar state usually has worse outcomes than isolated diabetic ketoacidosis. However, our data suggest that the combined diabetic ketoacidosis/hyperosmolar state has different clinical outcomes and needs to be recognized as different from isolated diabetic ketoacidosis and hyperosmolar state.

A study of the 28-day case-fatality rate of diabetic ketoacidosis and hyperosmolar state found that patients with diabetic ketoacidosis had a mortality rate of 6.10% in China, 11.8% in Korea, 17% in southern Taiwan, but only 3.9% in the United Kingdom and 2.4% in New Zealand ^16^. In our study, the mortality rate was 13.95%, similar to data found in the literature for diabetic ketoacidosis. The hyperosmolar state mortality rate was 18.83% in Taiwan, 17% in the USA, and 17% in Australia (8,9,16,17). Our mortality rate was higher in the diabetic ketoacidosis group than in the others. No deaths related to hyperglycemia were observed in the hyperosmolar state group, but in the combined diabetic ketoacidosis-hyperosmolar state group, there were 20 deaths (7,55%), which calls attention to the need to identify these patients.

Several factors have been suggested to be related to increased mortality. The hyperosmolar state may have a higher mortality rate depending on the serum osmolarity value: when the cutoff was 320 mOsm/L, the mortality rate was 4%. We could not confirm this finding in our population. However, invasive mechanical ventilation was associated with a higher occurrence of death, related to a higher severity of the disease presentation and a loss in body autoregulation. The risk of mortality also increased when pH < 7.3, probably due to a state of either respiratory or metabolic acidosis. In addition, it has been found that infections are the main precipitating cause of hyperglycemic crisis but might also be a cause of death in hyperglycemic crisis patients ^16^^,^^18^. In our study, it was the main precipitating cause, also associated with a higher risk of mortality, but it was not statistically significant.

Emphasizing the importance of combined states, we had more of these events than other crises. Our population consisted of 265 (83.5%) diabetic ketoacidosis/hyperosmolar state events, 43 (13.5%) diabetic ketoacidosis events, and only 9 (3%) hyperosmolar state events. According to the type of crisis, our frequency is different from that in the literature reports where diabetic ketoacidosis is most frequent using the same criteria. This discrepancy could be linked to type 2 diabetes mellitus predominance in our study (85.8%). In addition, the value of HbA1c obtained at the time of the crisis suggests that even those who admitted being adherent to the therapy had poor ambulatory glycemic control. It could also be related to the fact that the Colombian health care system has administrative and financial barriers, especially for the poorest, that tend to reduce access to the health care system ^19^ and may be aggravated by problems in adherence to treatment, education, economic situation, drug supply, or myths about the disease within the population.

**Figure 2:**
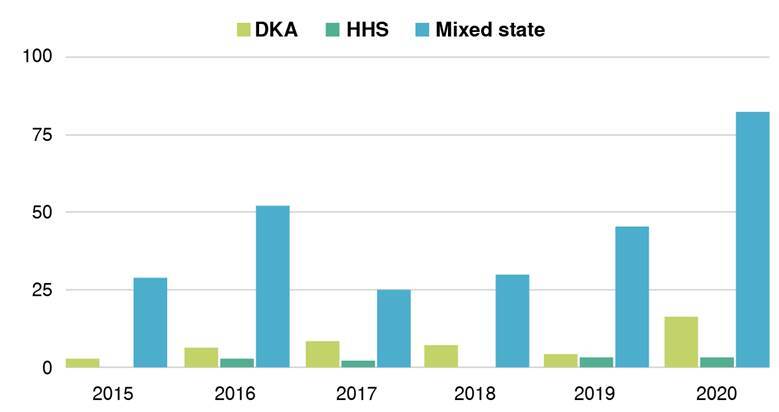
Prevalence of hyperglycemic crises per year

DKA: Diabetic ketoacidosis; HHS: Hyperosmolar hyperglycemic state.

In general hospitals, admissions for hyperglycemic crises have increased over the last few years ^20^. One of the most relevant data found during this research was that the number of hyperglycemic crises in 2020 increased dramatically, by approximately 50.98% compared to the previous year (figure 2), a situation we strongly believe is because of two main reasons. First, the appearance of the worldwide pandemic generated by the SARS- CoV-2 virus led to inadequate follow-up of patients and increased the probability of presenting this type of crisis; second, during this time, our institution increased their capacity to care for patients, opening the possibility for attending more. However, our hospital is also a referral institution in southwestern Colombia.

Kitabchi *et al*., in consensus with the American Diabetes Association, reported different precipitating factors of hyperglycemic crises. The two main factors were poor adherence to insulin treatment and concomitant infections. Other precipitating entities have been described, such as pancreatitis, acute myocardial infarction, stroke, trauma, and substance abuse. Diseases such as stroke and acute myocardial infarction probably trigger this complication by releasing counterregulatory hormones, leading to severe dehydration ^21^. These causes described in the literature are consistent with our results, where the main precipitating cause was infections, followed by poor adherence to treatment.

Regarding hospital stay length, we found an average of four days, with diabetic ketoacidosis cases having the longest average stay, at six days, shorter in contrast to the data from another hospital in Colombia where the hospital stay length for diabetic ketoacidosis was on average 18 ± 16 days, the longest of all groups ^15^. Another study in Spain showed a median hospitalization length of eight days for patients with diabetic ketoacidosis, most of them having a severe form of the disease ^22^. This fact explains the short hospital stays of the patients in our institution because we have a specific team of specialists attending to these crises in an optimal window of time. As the evidence showed, a specialized diabetes team helped to reduce hospital stay ^23^.

In conclusion, the combined diabetic ketoacidosis/hyperosmolar state was the most frequent cause of death related to the hyperglycemic crisis. Factors like invasive mechanical ventilation and a pH < 7.3 increased mortality. We propose including the combined diabetic ketoacidosis/hyperosmolar state in the hyperglycemic crisis classification.

Our study has limitations. It is a retrospective, observational study, and due to its nature, it was designed to examine the epidemiological features and factors associated with hyperglycemic crises, not to establish a causal relationship between any factor(s) and these events.
